# Taking Impression and Bites

**Published:** 1902-03-15

**Authors:** W. H. Stowe


### TAKING IMPRESSIONS AND BITES.

BY DR. W. H. STOWE.

It is a most lamentable fact that among the great majority of dentists the most important operations connected with the successful making of artificial teeth, viz.: taking the impression and "bite," are so slighted as to cause surprise that misfits are not universal and that the plates made from such poor foundations give the wearer any satisfaction.

Having for many years been in a position to see the models upon which thousands of plates were constructed, I feel justified in making the statement that not one in five is a correct representation of the mouth from which it was taken, being imperfect in some points, often important ones, but used with the supposition that a little judicious trimming of the model will remedy these defects.

The majority of impressions are taken hurriedly and in the manner that will be the easiest for the moment, without regard for the fact that a few minutes spent at that time to secure a perfect impression and bite will be more than made up by the ease and satisfaction with which the completed denture can be inserted.

It is not the purpose of this article to discuss the relative merits of the different impression materials, although the writer has very decided views on the subject, but to attempt to show the necessity of starting in the construction of a plate of any description with a model which shall be an exact duplicate, as nearly as it can be, of the mouth it represents, whether the impression has been taken with modelling composition, wax, or plaster, or a combination of these materials. Having obtained a perfect impression, the model should be made, and then carefully compared with the mouth, and trimmed and scraped in such a manner as may be deemed necessary by a careful diagnosis of the mouth. Then, many dentists have gotten into a slip-shod way of taking the bite—the result being that when the stage of arranging the teeth is reached, it becomes a matter of pure guesswork to remember what length and fulness they should be made. Don't hurry! If you have secured a perfect model, devote such time as may be necessary to obtain a "bite" that shall indicate every point you need to know in arranging the teeth. Length of teeth, amount of overlap, fulness of gum, extent the teeth or gums will show in laughing—all these important points can be shown as plainly as in the completed case, if sufficient time and care are taken at this stage in the construction of the case.

In partial cases requiring but a few teeth, the bite may be best obtained by placing in the spaces left by the missing teeth small pieces of softened bees' wax, and allowing the patient to bring the teeth together, taking care that they come together correctly. These pieces of wax can then be removed, trimmed to proper length, and placed on the model, and will show all that is necessary. In cases requiring four or more teeth it is best to make a trial plate of such material as may be preferred, and obtain the bite in wax attached to this plate. This wax should show not only the antagonizing teeth in their proper relative position, but should be so trimmed as to indicate the length the teeth are wanted, how much they are to be allowed to lap over the opposing teeth, and the fulness required.

By proceeding in this manner, all the elements of chance are eliminated, and the work not only proceeds with much less trouble, but the case when finished will prove satisfactory to dentist and patient alike, and this branch of dentistry, so despised by many, becomes, if not a pleasure, much less annoying than when done by the "luck" or "guess" process.—*Dental News*.

